# Nurses’ perceptions of patient safety competency: A cross-sectional study of relationships with occurrence and reporting of adverse events

**DOI:** 10.1371/journal.pone.0297185

**Published:** 2024-01-25

**Authors:** Edris Kakemam, Ahmed Hassan Albelbeisi, Mahtab Rouzbahani, Mehdi Gharakhani, Hamideh Zahedi, Roohangiz Taheri

**Affiliations:** 1 Clinical Research Development Unit of Tabriz Valiasr Hospital, Tabriz University of Medical Sciences, Tabriz, Iran; 2 Health Professions College, Al- Israa University, Gaza Strip, Palestine; 3 Department of Health Management and Economics, School of Public Health, Tehran University of Medical Sciences, Tehran, Iran; 4 Student Research Committee, Nursing and Midwifery Faculty, Tabriz University of Medical Sciences, Tabriz, Iran; 5 Department Health Services Management, School of Health, Qazvin University of Medical Sciences, Qazvin, Iran; King Saud University Medical City, SAUDI ARABIA

## Abstract

Although, strengthening patient safety competencies in nursing has been emphasized for enhancing quality care and patient safety. However, little is known about the association of nurses’ perceptions of patient safety competency with adverse nurse outcomes in Iranian hospitals. This study aimed to measure nurses’ levels of patient safety competency in the hospitals of Iran and examines the relationship between patient safety competency with the occurrence and reporting of adverse events (AEs). This cross-sectional research was applied in eight teaching hospitals in Tehran, Iran, between August and December 2021. A sample of 511 nurses was randomly selected using the table of random numbers. The validated Patient Safety Competency Self-Evaluation questionnaire was used. Furthermore, two questions were used to measure the incidence and reporting of AEs. Data analysis was performed using descriptive statistics, independent t-tests, and two binary logistic regression models through SPSS version 24.0. The mean patient safety competency score was 3.34 (SD = 0.74) out of 5.0; 41.5% of nurses rated their patient safety competency as less than 3. Among subscales, “skills of patient safety” scores were the highest, and “knowledge of patient safety” scores were the lowest. Nurses with higher ***Knowledge*** and ***Attitude*** scores were less likely to experience the occurrence of AEs (OR = 1.50 and OR = 0.58, respectively). Regarding AEs reporting, nurses with higher ***Skill*** and ***Attitude*** scores were 2.84 and 1.67 times, respectively, more likely to report AEs (OR = 2.84 and OR = 3.44, respectively). Our results provide evidence that enhancing PSC leads to reduced incidence of AEs and increased nurses’ performance in reporting. Therefore, it is recommended that managers of hospitals should enhance the patient safety competency of nurses in incidents and reporting of patient safety adverse outcomes through quality expansion and training. Additionally, researchers should carry out further research to confirm the findings of the current study and identify interventions that would strengthen patient safety competencies and reduce the occurrence of AEs, and rise their reporting among nurses.

## Introduction

In the last years, the safety and quality of care in the healthcare system have been raised as main concerns [1]. Adverse events (AEs) are one of the most serious issues faced by health systems worldwide and seriously threaten patient safety and quality of care in hospitals [2]. AEs include a preventable error or a near miss that occurs while providing care for patients, which harms the patient and is unrelated to the underlying disease [2, 3]. Worldwide, risky care due to AEs is reported to be one of the fifteenth leading causes of death and infirmity; about 50% of the incidence of AEs can be prevented [4, 5]. In developing countries, the potential AEs are far above that in developed nations. For instance, in the Eastern Mediterranean Region, an estimated 4.4 million AEs occur annually, with 18% of inpatient admissions linked to AEs which are frequently preventable and result in high rates of mortality and lifelong disability [6]. A systematic review reported the prevalence of AEs between 10 to 80% in Iran [7]. Furthermore, the finding study conducted in Iran demonstrated that the occurrence of AEs varied from 51.2–63.0% in the past year among nurses [2]. Another study conducted among nurses in Iranian teaching hospitals showed that 67.5% reported having experienced AEs, leading to patient damage, and 65.2% said they had appropriate AEs reporting [8].

Nurses are the main body of health service providers, and quality implementations rely on them. They are essential to patient safety because they are accountable for ongoing and immediate care [9, 10]. Patient safety is directly linked to the safety culture among nurses. A robust safety culture is defined by management’s dedication to fostering a learning environment from errors, enhancing patient safety, promoting and practicing teamwork, recognizing potential hazards, implementing a system for reporting and analyzing adverse events within the hospital setting, and continuously evaluating the safety culture [11]. Findings from Aiken’s study show that many nurses do not feel comfortable reporting errors. Therefore, enhanced focus on cultivating a non-punitive culture and enhancing work environments to boost nurses’ trust in management can facilitate improved adverse event reporting, which is pivotal for organizational learning in the realm of patient harm prevention [12]. One study in Iran showed that to maintain and enhance patient safety in Iran’s healthcare system, it is essential to establish an effective error reporting system that is based on a "no-blame" culture. Moreover, Iranian hospitals should prioritize fostering a culture of patient safety and reporting medical errors [13].

Over the past decade, many interventions have been used to mitigate AEs and improve patient safety [2–4]. Among these strategies, improving patient safety competency is vital in timely reporting and adopting preventive measures for AEs and ensuring patient safety [14, 15]. As one of the largest groups of medical workers, nurses play a vital role in providing safe and quality patient care. Therefore, nurses must be equipped with PSC in their studentship or working periods [16]. Patient safety competency encompasses the knowledge, skills, and attitudes about patient safety, which are deemed essential for healthcare professionals to effectively protect patients from harm [17, 18].

A growing body of evidence has examined levels of knowledge, skills and attitudes regarding patient safety in healthcare professionals [14, 15]. For instance, a study on psychiatric ward nurses’ patient safety knowledge found they rated themselves as low [19]. Another study involving Korean nurses revealed moderate levels of patient safety competency [15]. In Iran, to ensure that the knowledge and skills of nursing graduates meet the requirements for providing safe and high-quality services, the nursing curriculum has been modified several times over the past decades. The latest changes were applied in 2011 and were guided by several principles, including student-centered education, competency-based education, community-oriented education, problem-based learning, and evidence-based nursing. Although the basic principles of patient safety are taught to students by nursing instructors throughout the program, as well as some courses were held by hospitals for their clinical staff, the absence of dedicated patient safety education in healthcare curricula leads to an inadequacy in students’ preparedness for incorporating safety principles into patient care delivery before their transition into clinical practice settings [20].

Several empirical investigations have explored the association between patient safety competency and AEs among nurses in other nations [10, 21]. Although the researchers have previously conducted studies on other strategies including patient safety culture, teamwork and systems thinking, and adverse events among Iranian nurses [2, 22, 23]. However, to our knowledge, this is the first study that has examined the relationship between nurses’ perception of patient safety competency and AEs in Iran. Estimating the prevalence of AEs, timely reporting and identifying, and analyzing the leading factors are key preventive strategies [24]. Finally, considering the ever-changing nature of healthcare systems, changes in patient safety practices, and the need for continuous improvement in patient care carrying out new studies to provide valuable insights and contribute to this field’s expanding body of knowledge are necessary. Therefore, the current research sought the following purposes: (1) to measure the levels of nurses’ patient safety competency and the occurrence and reporting of AEs, and (2) to examine the relationship between nurses’ levels of patient safety competency and the occurrence and reporting of AEs.

## Method

### Study design

A descriptive cross-sectional research was applied in eight teaching hospitals in Tehran, the capital of Iran, between August and December 2021.

### Participants and setting

The target population consisted of all clinical nurses who provided care to patients in eight hospitals affiliated with Iran University of Medical Sciences, Tehran. Eligibility for study participation was restricted to clinical nurses who had provided direct patient care in clinical settings for a minimum of six months. Individuals who were nursing students undergoing training at the hospital held managerial or administrative positions or had less than six months of nursing experience were excluded.

To determine the appropriate sample size for the study, we used the Cochran formula [25], It was determined that a sample size of 422 nurses would be needed, with a 4% margin of error and a 95% confidence level. To allow for a non-response rate of 20%, we increased the sample size to 528. We assigned each hospital a quota based on the number of nursing staff and recruited nurses from each ward using a simple random sampling technique.

### Measures

#### Socio-demographic characteristics

Socio-demographic characteristics of respondents included age, gender, marital status, education level, experiences in nursing, clinical department, job position, and type of employment.

#### Adverse events

Adverse Events were defined as preventable errors or near misses, such as a pressure ulcer, patient fall, medication error, infusion or transfusion reaction, and surgical wound infection. The occurrence and reporting of AEs were measured by asking two questions that were used in previous studies [3, 22, 23]: ‘‘Nurses were asked to indicate whether they had experienced clinical errors in the last six months?” and " whether they then reported the errors to their managers or the patient safety department?”. Items were rated on a 5-point Likert scale (0 = never, 1 = rarely, 2 = sometimes, 3 = usually, and 4 = always). To assess the association between PSC and AEs incidence, the answers were dichotomized by codifying ‘never’ as ‘never experienced AEs’ and the other answers as ‘experience with AEs’ concerning the incidence of AEs. Likewise, regarding reporting the AE to the supervisor or patient safety department, responses "always" or "usually" were considered appropriate for reporting AEs. The remaining responses ("sometimes", "rarely", and "never") were considered as "inappropriate" performance [3, 22, 23]. The face validity of the tool was confirmed by experts in patient safety and nursing. Also, these items demonstrated a high internal consistency and reliability (Cronbach’s α = 0.75).

#### Patient safety competency

Nurses’ PSC were measured using the Patient Safety Competency Self-Evaluation (PSCSE) tool developed by Lee et al [17]. This is a validated 41-item survey assessing self-reported patient safety competencies that includes three dimensions, namely Knowledge (six items), Skill (21 items), and Attitude (14 items). A 5-point Likert scale was used to score the nurses’ responses: knowledge (1 point: not knowledgeable, 5 points: very knowledgeable), skills (1 point: very uncomfortable, 5 points: very comfortable), and attitudes (1 point: strongly disagree, 5 points: strongly agree). Two items related to the attitude, including 8 and 9, were reversed. We measured the total patient safety competency score by averaging the average of the three domains. The PSC scores range from 1 to 5, with higher scores indicating greater competency. A score above 3 suggests nurses are confident in patient safety competency [15]. In Lee et al.’s study, Cronbach’s α for the scale was 0.91 [17].

We obtained the developers’ permission to translate and validate the questionnaire. Then, a cross-cultural guiding process was used in translating the English version into Farsi [26]. The content validity of this questionnaire was checked by a panel of experts consisting of seven members of the Faculty of Nursing and Midwifery University of Iran Medical Sciences. This study used internal consistency and reliability (test-retest) to calculate the reliability. To conduct the test-retest, the questionnaire was given to 35 nursing staff eligible to enter the study in two stages with a time interval of approximately two weeks, and interclass correlation coefficients were calculated for all questions and the entire questionnaire. According to Cronbach’s alpha method, reliability was obtained for the dimensions of knowledge, attitude, and skill, respectively, 0.82, 0.76, 0.95, and 0.95 for all dimensions. The data of 35 nurses were not considered in the primary sampling.

#### Data collection

Data collection commenced on August 5, 2021, and concluded on December 28, 2021. Before data collection, the study protocol received ethical approval from the Iran University of Medical Sciences Ethics Committee. Subsequently, two researchers conducted hospital visits on various weekdays during morning, evening, and night shifts to gather the necessary data. Questionnaires were distributed, completed, and collected by the researchers. Adhering to ethical principles, nurses voluntarily participated in the study and completed the questionnaires. Following an explanation of the study’s objectives, participants were assured of response confidentiality, and informed consent was obtained from all nurses. Subsequently, questionnaires were distributed to nurses in person and subsequently collected.

#### Bias

We collected the data in three shifts: morning, afternoon, and night. Participation in the survey was voluntary, and the confidentiality of the data was ensured. Moreover, we provided some items reversely in a questionnaire to reduce or prevent response bias. Additionally, nurses were informed of the aims of the study and how to complete the questionnaire and gave respondents sufficient time to complete the questionnaire.

#### Ethics consideration

Ethical approval for this study was granted by the Research Ethics Committee of Iran University of Medical Sciences under the ethics code (NO IR.IUMS.REC.1397.1137). Authorization to conduct the study was obtained from Iran University of Medical Sciences. All participants provided written informed consent, which was reviewed and approved by the Research Ethics Committee. Participants retained the absolute right to decline or withdraw from participation, and the confidentiality of all participant information was strictly maintained. The study adhered to the principles outlined in the Declaration of Helsinki, 2008.

#### Data analysis

Analysis was completed using SPSS version 24.0. Descriptive statistics (frequency distributions, mean, and standard deviation) were used. Independent t-tests were employed to assess differences in PSC scores between nurses demonstrating appropriate and inappropriate AE reporting practices, as well as between nurses with and without AEs. A binary logistic regression model was utilized to investigate the association between PSC and the occurrence and reporting of AEs. The model was adjusted for potential confounding variables, and the odds ratios (ORs) and 95% confidence intervals (CIs) for the variables were calculated. Statistical significance was set at a p-value < 0.05.

## Results

### Socio-demographic characteristics of the participants

Of 528 nurses, 511 (96.8% return rate) completed the questionnaires. Table 1 displays the characteristics of the participants who participated in the study. More than half (54.8%) of nurses aged 31 or above. Less than half of the nurses (44.6%) were male. Only 11.2% had a master or Ph.D. degree, and about 62% were married. The majority (61.4%) of participating nurses have nursing experience of fewer than five years. 94.5% are working in clinical nurse roles. Regarding the clinical department where nurses work, the vast majority (78.1%) worked in general departments. Most (83.2%) of participating nurses have permanent or contract jobs.

**Table 1:** Socio-demographic characteristics of the participants and patient safety competency scores.

Variable	N (%)	Patient safety competency	P-value
		Mean	
**Age (years)**			
*23–30*	231 (45.2)	3.34	<0.001
*31–40*	261 (51.1)	3.15	
*>40*	19 (3.7)	3.61	
**Gender**			
*Male*	228 (44.6)	3.15	<0.001
*Female*	283 (55.4)	3.49	
**Marital status**			
*Single*	192 (37.6)	3.39	0.217
*Married*	319 (62.4)	3.31	
**Education level**			
*Bachelor*	454 (88.8)	3.38	0.004
*Master or PhD*	57 (11.2)	3.08	
**Experience in nursing (years)**			
*1–5*	314 (61.4)	3.25	<0.001
*6–10*	142 (27.8)	3.29	
*>10*	55 (10.8)	3.99	
**Clinical department**			
*ICU*	54 (10.6)	3.57	0.011
*Emergency*	58 (11.3)	3.48	
*General Wards*	399 (78.1)	3.29	
**Job position**			
*Nurse*	483 (94.5)	3.32	0.021
*Manager*	28 (5.5)	3.68	
**Type of employment**			
*Permanent*	191 (37.4)	3.52	<0.001
*Contract*	234 (45.8)	3.16	
*Bonded*	86 (16.8)	3.43	

### Patient safety competency

The mean score for overall PSC and its sub-scales are shown in Table 2. The mean overall PSC was 3.34 (SD = 0.74), and 41% of nurses rated their competency less than 3.0, indicating that they were not confident in patient safety practice. At a subscale level, the “Skills” scores were the highest, with a mean of 3.49 (SD = 0.88) out of 5, with 43.06% of the respondents having skills score of less than 3.0. One item with the highest scores was “Administer drug to the patient according to medication policies for safe care”, with a mean of (Mean± SD = 3.68±1.23). The item “Disclose an error to a manager of the unit or patient safety department” had the lowest score (Mean± SD = 3.31±1.24). The mean “Attitude” score was 3.48±0.88. The percentage of nurses with ratings less than 3.0 was 37.77%. One of the items with the highest score was ‘A standardized procedure minimizes risks associated with handoff’ (Mean± SD = 3.62±1.22), while the items with the lowest scores included ‘Error disclosing (Mean± SD = 3.16±1.42). The mean score for “Knowledge” was 3.06±0.78, with 60% of the respondents having a knowledge score of less than 3.0. At items level, the “Describe role of human factors in assuring safety” and “Explain how authority gradients (horizontal, vertical) influence teamwork and patient safety” scores were the highest, both with a mean of 3.17 (SD = 1.02 and 1.13, respectively) and the ‘Describe factors that create a culture of safety score was the lowest, with a mean of 2.81 (SD = 1.16).

**Table 2:** Levels of patient safety competencies.

Scales/items	n^a^	%^a^	Mean	SD	α
**Overall patient safety competency**	**299**	**58.51**	**3.34**	**0.74**	**95.3**
***Attitude***	**318**	**62.23**	**3.48**	**0.80**	**75.7**
Errors are preventable	278	54.4	3.31	1.28	
Making an effort to improve patient safety	302	59.1	3.54	1.17	
Intolerance of uncertainty in patient care	278	54.4	3.40	1.31	
To share information about medical errors	309	60.5	3.56	1.20	
To priorities patient safety for nurses	290	59.7	3.56	1.26	
Error reporting	219	56.7	3.56	1.30	
Error disclosing	136	42.9	3.16	1.42	
If there is no harm to the patient there is no need to report an error	269	52.7	3.42	1.34	
If I saw an error, I would keep it to myself	273	53.5	3.50	1.37	
Using technology and information management tools to support safe processes of care	273	53.4	3.43	1.24	
To play key role in preventing errors.	298	58.3	3.60	1.32	
Value nurses’ involvement in design, selection, implementation, and evaluation of information technologies to support patient care	311	60.9	3.60	1.18	
A standardized procedure minimizes risks associated with handoff (e.g., transfer, shifts) within disciplines and across transitions in care	304	59.5	3.62	1.22	
***Knowledge***	**198**	**38.7**	**3.06**	**0.78**	**82.2**
Describe factors that create a culture of safety	126	24.6	2.81	1.16	
Describe role of human factors in assuring safety	184	36.0	3.17	1.02	
Distinguish among errors, adverse events, near misses, and hazards	176	34.4	3.06	1.15	
Describe processes used in analyzing causes of error	173	33.9	3.09	.98	
Describe the impact (benefits and limitations) of technology and information management care	153	29.9	3.04	.99	
Explain how authority gradients (horizontal, vertical) influence teamwork and patient safety	199	38.9	3.17	1.13	
***Skills***	**291**	**56.94**	**3.49**	**0.88**	**95.3**
Report errors using an organizational error reporting system	257	50.3	3.33	1.30	
Accurately enter an error report	262	51.3	3.40	1.14	
Analyze a case to find the causes of an error	259	50.7	3.38	1.25	
Support and advise a peer who must decide how to respond to an error	268	52.4	3.49	1.14	
Disclose an error to a managers of unit or patient safety department	243	47.6	3.31	1.24	
Communicate observations or concerns related to hazards or errors with health care professionals	268	52.4	3.44	1.18	
Communicate observations or concerns related to hazards or errors with an affected patient s and his or her family	275	53.8	3.48	1.17	
Locate evidence reports related to clinical practice topics and guidelines to define uncertainty in nursing care	274	53.6	3.43	1.16	
Use high quality electronic sources of health care information	269	52.7	3.38	1.24	
Use technology and information management tools to support safe processes of care	248	48.5	3.43	1.24	
Prevent and manage pressure ulcers	277	54.2	3.45	1.33	
Practice hand hygiene to prevent infection	291	57.0	3.55	1.27	
Us e falls risk assessment tool to prevent falls	303	59.3	3.63	1.29	
Give a blood transfusion according to transfusion policies for safe care	307	60.0	3.62	1.26	
Administer drug to patient according to medication policies for safe care	308	60.3	3.68	1.23	
Follow communication practices that minimize risks associated with hand offs between and among providers and across transitions in care.	299	58.5	3.53	1.26	
Document hand off communication according to institutional policies.	287	56.2	3.59	1.18	
Use standard infection control precautions for all patient encounters and other transmission precautions as appropriate	275	53.8	3.49	1.25	
Use appropriate personal protective equipment	292	57.2	3.60	1.20	
Apply aseptic technique when inserting invasive devices as appropriate for patient care procedures	282	55.2	3.53	1.26	
Check patient’s identity accurately	288	56.4	3.55	1.26	

^a^Numbers and proportions of the nurses who rated their patient safety competency above 3.0, respectively.

The bolds indicate the scores for overall scale and subscales.

The mean differences in PSC scores according to the general characteristics of study participants are presented in Table 1. Respondents aged between 31 and 40 years rated their PSC lower than the others (p<0.001). Significant differences were observed in PSC, with female nurses (p<0.001) and those who had higher education levels (p<0.001) having significantly higher PSC scores. Nurses working in ICUs rated their patient safety competency as higher than the others (p = 011). There is a statistically significant difference in terms of PSC level based on length of clinical experience (p < .001), with significantly higher levels of PSC observed in nurses who had more than ten years of experience, compared to both those with 5–10 years and less than five years of experience. The PSC scores of the nurse managers were higher than those of the staff nurses (p = 0.021). The mean PSC among participants with contract employment was significantly lower than those with permanent and bonded employment (p < .001).

### Association between patient safety competencies and AEs occurrence and reporting

The majority of nurses (345; 67.5%) reported that they had experienced AEs and 65.1% (333) stated that they had reported AEs to their supervisors or the patient safety department. Table 3 compares scores for the PSC dimensions (Skills, Attitudes, and Knowledge) by AEs occurrence and reporting. The level of patient safety competency among nurses who experienced AEs was significantly lower across all subscales than those with no AEs experience (p < .001). Also, nurses who had appropriately reported AEs had higher patient safety competencies scores than those who had not (p < .001).

**Table 3:** Comparison of scores of patient safety competencies by AEs occurrence and reporting.

Variables	Nor experience of AEs (n = 166)	Experience of AEs (n = 345)	*P*	Inappropriate AEs reporting (n = 178)	Appropriate AEs reporting (n = 333)	*P*
	Mean (SD)	Mean (SD)		Mean (SD)	Mean (SD)	
**Skills**	3.70 (0.96)	3.38 (0.81)	<0.001	2.84 (0.62)	3.83 (0.80)	<0.010
**Attitudes**	3.72 (0.84)	3.37 (0.75)	<0.001	2.88 (0.55)	3.80 (0.72)	<0.001
**Knowledge**	3.16 (0.83)	3.01 (0.75)	0.039	2.57 (0.65)	3.32 (0.72)	<0.001

Table 4 shows the results of multiple logistic regression analyses for the occurrence of AEs and events reporting. The results display that female nurses were more likely to experience AEs than male nurses [odds ratio (OR) = 1.63; 95% CI = 1.07–2.47]. Furthermore, married nurses were 1.86 times more likely to report AEs to their manager or patient safety department than single nurses [odds ratio (OR) = 1.86; 95% CI = 1.05–3.31]. Regarding the occurrence of AEs, nurses with a higher ***Knowledge*** score were 1.50 times less likely to experience the occurrence of AEs (OR = 1.50; 95% CI = 1.02–2.20). Moreover, nurses with more positive ***Attitudes*** toward patient safety were less likely to experience adverse events (OR = 0.58; 95% CI = 0.36–0.95). Regarding AE reporting, nurses with higher ***Skill*** and ***Attitude*** scores were 2.84 and 1.67 times more likely to report AEs to their supervisor or patient safety department.

**Table 4:** Logistic regression analysis results for appropriate events reporting and the occurrence of adverse events.

Variable	Occurrence of AEs		Appropriate AEs reporting	
	OR [95% CI]	*P*	OR [95% CI]	*P*
**Age** (*Reference*: 23–30 years)				
31–40 years	0.85 [0.23–3.14]	0.81	0.38 [0.70–2.09]	0.26
>40 years	1.16 [0.37–3.67]	0.78	0.79 [0.17–3.66]	0.77
**Gender** (*Reference*: Male)	1.63 [1.07–2.47]	*0*.*02*	1.09 [0.66–1.78]	0.72
**Marital status** (*Reference*: Single)	0.90 [0.57–1.42]	0.66	1.86 [1.05–3.31]	*0*.*03*
**Educational level** (*Reference*: Bachelor)	0.85 [0.41–1.77]	0.67	1.09 [0.50–2.38]	0.82
**Experiences in nursing** (*Reference*: 1–5 years)				
*6–10 years*	1.93 [0.82–4.53]	0.12	1.28 [0.37–4.34]	0.68
*>10 years*	1.56 [0.74–3.25]	0.23	0.96 [0.30–3.05]	0.94
**Clinical department** (*Reference*: ICU)				
*Emergency department*	0.59 [0.31–1.11]	0.10	1.53 [0.68–3.40]	0.29
*General wards*	1.04 [0.55–1.94]	0.90	1.18 [0.53–2.61]	0.66
**Job position** (*Reference*: Nurse)	1.22 [0.45–3.30]	0.68	1.55 [0.41–5.85]	0.51
**Type of employment** (*Reference*: Permanent)				
*Contract*	0.69 [0.33–1.42]	0.31	1.33 [0.55–3.23]	0.52
*Bonded*	1.08 [0.60–1.95]	0.77	1.03 [0.50–2.13]	0.92
**Skill**	0.85 [0.57–1.25]	0.41	2.84 [1.67–4.83]	*<0*.*001*
**Knowledge**	1.50 [1.02–2.20]	*0*.*03*	1.41 [0.88–2.27]	0.14
**Attitude**	0.58 [0.36–0.95]	*0*.*03*	3.44 [1.87–6.35]	*<0*.*001*
Nagelkerke R Square	.106		.458	

## Discussion

Findings from this study have provided new evidence of the level of nurses’ perceptions of PSC and the association between PSC and AEs occurrence and reporting. Although the results of our study demonstrated that nurses’ perception of overall PSC was 3.44 out of 5.0, 41% of nurses rated their competency less than 3.0, indicating that they were not equipped with enough competency for patient safety. Among subscales, more than 60% of nurses did not know enough about patient safety. This could be attributed to most practicing nurses receiving little or no formal education at university to prepare them for patient safety issues. In addition, nurses do not have enough time to participate in patient safety training courses in the workplace due to heavy workloads and nurse shortages. Therefore, they need systematic training regarding patient safety. The overall PSC scores estimated in our research were lower than those of previously found scores by this scale in Iran [8] and South Korea [27]. Regarding the three dimensions of PSC, the outcomes showed that knowledge about patient safety received the lowest score at 3.06 out of 5, consistent with the findings of a previous study conducted in Iran [8]. Another research conducted among emergency nurses in Iran showed that the knowledge, skills, and attitudes toward patient safety were lower than our results [14]. A study conducted in South Korea among nursing school clinical instructors and hospital nurses showed that both groups scored higher than Iranian nurses in knowledge, skills, and attitudes and that hospital nurses had significantly higher mean scores than clinical instructors in terms of knowledge [28].

The current outcomes can be clarified by cultural differences in patient safety teaching worldwide, and the gap between nurses in different countries’ results could be related to the fact that many countries started educating patient safety concepts many years ago compared to other countries [29]. Furthermore, the other prominent reasons for these contradictory findings could be due to the difference in organization-related parameters, such as discrepancies in organizational culture, organizational structure and organizational policies and individual parameters, including patient safety education and the concept of PSC and PSC culture, may have an effect on the PSC perception by nurses. The current findings showed a need to strengthen Iranian nurses in the three dimensions of PSC. Therefore, strategies, including training courses and patient safety improvement interventions, are needed to enhance the level of PSC among nurses with low PSC levels. Moreover, nursing schools should integrate patient safety education into formal nursing curricula.

The findings showed that 67% of nurses experienced the occurrence of AEs in the past six months. These findings were consistent with a previous study conducted in Iran [30] which showed the prevalence of AEs was between 33.1 and 51.1%. Song et al. in China revealed that the prevalence of AEs was 13.9% of operating room nurses [31]. It has approaches recommended such as quality assurance/peer review, evaluation of safety behavior, training and procedures, and root cause analysis to prevent AEs [32]. Regarding the reporting of AEs, 65% of nurses had reported AEs to their supervisors or the patient safety department, which was higher than those of nurses and physicians (55.5%) in Saudi Arabia [33] and among Korean nurses (51.4% and 53.0%) [3, 34]. Studies showed timely and reliable reporting of adverse events is critical for organizational learning and the improvement of more dependable healthcare systems [24, 35]. Although the rate of AEs reporting was appropriate compared to other studies, effective interventions and strategies should still be considered to improve error reporting performance among Iranian nurses. Hospital managers can implement strategies such as voluntary error reporting systems and establishing a positive environment and non-blame culture.

The findings indicated that nurses with higher patient safety competencies scores, including knowledge and attitude, were less likely to experience the occurrence of AEs. Moreover, nurses with higher skills and attitudes are more likely to report AEs to their managers or patient safety departments. These results were in line with a previous study conducted in South Korea that had reported low perceived patient safety competencies were correlated with AEs [10]. Likewise, a prior study among nurses in South Korea demonstrated that higher mean PSC was significantly correlated with declines in the occurrence and reporting of AEs [21]. A recent study reported that weak PSC levels in emergency department nurses had a negative correlation with the reporting of patient safety AEs [27]. According to Alves and Guirardello, creating a conducive work environment is a key factor in the roles of organizational leaders and nursing departments concerning patient safety and professional practices, which is consistent with our study outcomes [36]. Hwang also found that nurses with high patient safety nursing perceived patient safety climate more positively and emphasized that teamwork should be implemented, which increases nurses’ patient safety competence [15]. Furthermore, another study in Korea showed that higher mean scores for “understanding human and environmental factors” in PSC were significantly correlated with reduced incidences of drug errors and urinary tract infections. Nurses with higher safety competencies are expected to be dedicated to patient safety by supporting a positive safety culture [37]. Therefore, the development and implementation of intervention programs that train and empower nurses’ hospital PSCs are necessary to reduce adverse effects. In addition, hospital administrators should strive to promote a non-punitive culture against AE events, as long as a healthy work environment, including a culture of patient safety, is compromised.

### Limitations of study

The current research has some limitations that need to be addressed. First, the survey was conducted only with nurses working in the capital of Iran, so the results may not apply to nurses working in other regions. Second, the research used a cross-sectional design, which may not be sufficient to determine the effect of patient safety competency on AEs. Future studies should consider using other designs such as longitudinal and intervention studies. Finally, the measurement of patient safety competency levels relied on self-administered questionnaires, which can be biased due to recall and social desirability. Therefore, future research should consider using more objective tools to measure the actual level of PSC among nurses.

### Implication for managers

The results highlighted a need to strengthen Iranian nurses in the three dimensions of PSC. Therefore, hospital managers and educators must ensure the development of the necessary competencies in nurses by developing a training package and holding courses. In addition, in-service continuing education programs should be implemented for nurses to enable them to provide clinically safe, high-quality care. Furthermore, continuing in-service education programs must be implemented for nurses to help them to provide clinically safe, high-quality care. Finally, hospital managers should strive to foster a non-punitive culture against AEs until healthy work environments, including a culture of patient safety, are compromised.

## Conclusion

The findings indicate that Iranian nurses’ PSC need to be strengthened. Furthermore, improving PSC can lead to a decrease in the incidence of AEs and an increase in nurses’ reporting performance. To achieve this, a structured training program should be developed and implemented to strengthen the PSC of nurses who are vulnerable and have low patient safety competency. Additionally, further research should be conducted to validate the results of this study and identify interventions that can strengthen patient safety competencies, reduce the occurrence of AEs, and encourage reporting among nurses.

## Supporting information

S1 File (SAV)
